# Activity of menin inhibitor ziftomenib (KO-539) as monotherapy or in combinations against AML cells with MLL1 rearrangement or mutant NPM1

**DOI:** 10.1038/s41375-022-01707-w

**Published:** 2022-09-23

**Authors:** Warren Fiskus, Naval Daver, Steffen Boettcher, Christopher P. Mill, Koji Sasaki, Christine E. Birdwell, John A. Davis, Kaberi Das, Koichi Takahashi, Tapan M. Kadia, Courtney D. DiNardo, Francis Burrows, Sanam Loghavi, Joseph D. Khoury, Benjamin L. Ebert, Kapil N. Bhalla

**Affiliations:** 1 The University of Texas M.D. Anderson Cancer Center, Houston, TX 77030 USA; 2 University of Zurich and University Hospital Zurich, CH-8091 Zurich, Switzerland; 3 Kura Oncology, Inc., San Diego, CA 92130 USA; 4 Howard Hughes Medical Institute, Dana-Farber Cancer Institute, Boston, MA 02115 USA

**Keywords:** Acute myeloid leukaemia, Targeted therapies

## To the Editor:

In MLL1-rearranged AML (MLL1-r), the MLL1 fusion protein (MLL-FP) causes dysregulated expression of HOXA9, MEIS1, PBX3, MEF2C and CDK6 [1, 2]. HOXA9 and its co-factor MEIS1 are leukemogenic in myeloid stem progenitor cells [3–5]. Additionally, in AML with mtNPM1 (NPM1c), MLL1 is the main oncogenic regulator of HOXA9, MEIS1 and FLT3, promoting self-renewal of myeloid progenitor cells [2, 6, 7]. Orally bioavailable, investigational or clinical drug candidate Menin inhibitors (MIs) disrupt binding of Menin to its binding pocket in MLL1/2 and MLL1-FP, which reduces MLL1/2 and MLL1-FP binding to their targets, inhibits HOXA9/MEIS1 activity, represses PBX3, MEF2C, FLT3 and CDK6, as well as induces differentiation and loss of survival of AML with MLL1-r or with mutant (mt)-NPM1 [2, 8–10]. In early clinical trials, monotherapy with MI is well tolerated and has achieved objective remissions in patients with previously treated relapsed/refractory AML harboring MLL1-r or NPM1c [2, 11]. However, most patients either fail to respond or eventually relapse [11]. Therefore, there is a need to investigate the activity of additional MIs and MI-based combinations that may exhibit superior activity and prevent or abrogate MI-resistance in AML cells with MLL1-r or mtNPM1.

**Fig. 1:**
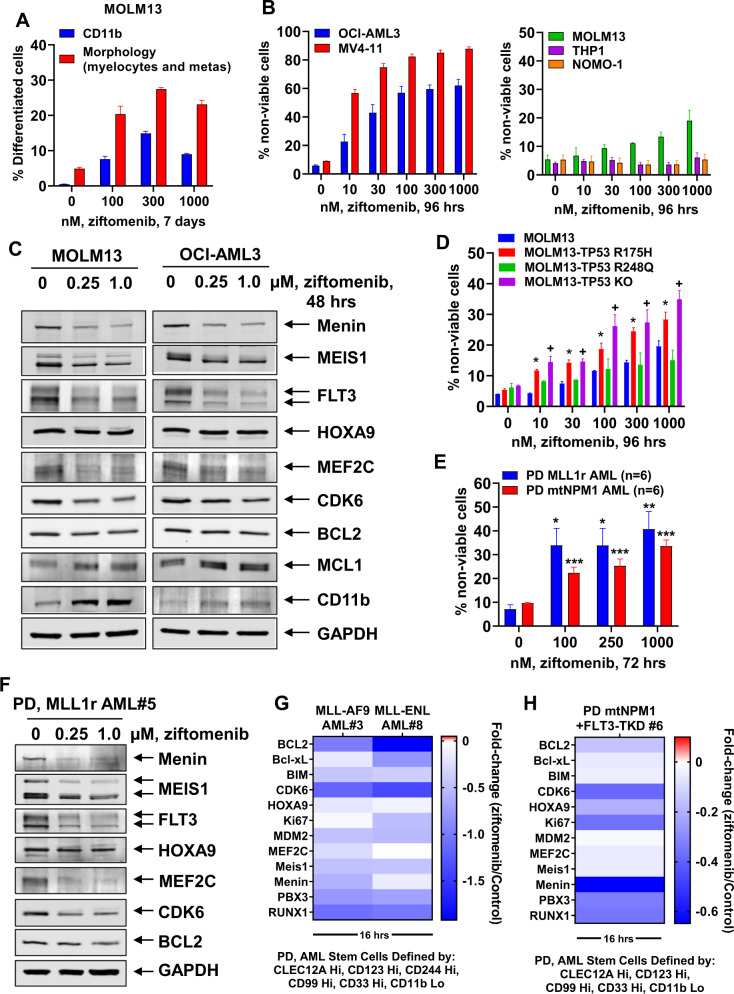
Treatment with Menin inhibitor ziftomenib depletes Menin expression, alters MLL1 target gene expressions in patient-derived AML stem cells defined by high expression of CLEC12A, CD123, CD99, and CD33 and induces differentiation and cell death of AML cells with MLL1 rearrangement or mutant NPM1. **A** MOLM13 cells were treated with the indicated concentrations of ziftomenib for 7 days. Following this, the % of CD11b-positive cells was determined by flow cytometry. Features of morphologic differentiation were assessed in cells cytospun onto glass slides and stained with hematoxylin and eosin. Columns; mean of three experiments ± S.E.M. **B** OCI-AML3, MV4-11, MOLM13, THP1 and NOMO-1 cells were treated with the indicated concentrations of ziftomenib for 96 h. At the end of treatment, cells were stained with To-Pro-3 iodide and the percentage of non-viable cells was determined by flow cytometry. Columns; mean of three experiments ± S.E.M. **C** Representative immunoblot analyses of Menin and MLL1 fusion target genes in MOLM13 and OCI-AML3 cells treated with the indicated concentrations of ziftomenib for 48 h. The expression of GAPDH in the lysates served as the loading control. **D** Isogenic MOLM13 cells with TP53 mutation (R175H or R248Q) or bi-allelic TP53 knockout (KO) were treated with the indicated concentrations of ziftomenib for 96 h. At the end of treatment, cells were stained with To-Pro-3 iodide and the percentage of non-viable cells was determined by flow cytometry. Columns; mean of three experiments ± S.E.M. *Cell death values significantly greater (*p* < 0.05) in MOLM13 TP53-R175H compared to MOLM13 (TP53 wt). ^+^Cell death values significantly greater (*p* < 0.05) in MOLM13 TP53-KO compared to MOLM13 (TP53 wt). **E** Patient-derived (PD) AML cells with MLL1 rearrangement or with mtNPM1 expression were treated with the indicated concentrations of ziftomenib for 72 h. Following this, the cells were stained with To-Pro-3 iodide and the percentage of non-viable cells was determined by flow cytometry. Columns; mean ± S.E.M for each type of AML. *p < 0.05, ***p < 0.005, relative to untreated cells, as determined by a two-tailed, unpaired *t*-test. **F** Patient-derived MLL1-rearranged AML cells were treated with the indicated concentrations of ziftomenib for 48 h. Total cell lysates were prepared and immunoblot analyses were conducted. The expression levels of GAPDH in the cell lysates served as the loading control. **G**, **H** Patient-derived MLL1-rearranged and mtNPM1-expressing AML cells were treated with 1 µM of ziftomenib for 16 h. Then, cells were incubated with cocktails of rare, heavy metal ion-tagged antibodies against extracellular and intracellular proteins. Mass cytometry (CyTOF) analysis was performed on the untreated and treated cells and the data were analyzed by Astrolabe. Protein expression alterations in ziftomenib-treated cells are shown as a fold-change, relative to the control cells in phenotypically defined AML stem cells.

Here, we determined that exposure for 4 days to the orally bioavailable, investigational, MI ziftomenib (KO-539) dose-dependently inhibited cell growth of MOLM13 (expressing MLL1-AF9) and OCI-AML3 cells (expressing mtNPM1, DNMT3A R882C and homozygous activating NRAS-Q61L mutations) (Supplementary Figs. S1A and S1B). Ziftomenib treatment for 7 days induced expression of the myeloid differentiation marker CD11b and increased morphologic features of differentiation (Fig. 1A, Supplementary Figs. S1C and S1D) [10]. Treatment with ziftomenib for 4 days dose-dependently induced loss of viability in OCI-AML3 and MV4-11 cells, but less so in MOLM13 and was ineffective in THP1 or NOMO-1 cells (Fig. 1B and Supplementary Figs. S1B–S1E). Ziftomenib treatment reduced mRNA levels of MEIS1, PBX3, MYC, FLT3, CDK6 and BCL2, while inducing mRNA levels of CD11b (Supplementary Figs. S2A and S2B). Notably, ziftomenib also reduced protein levels of Menin, as well as of MEIS1, FLT3, MEF2C, CDK6, and BCL2, without affecting HOXA9 protein levels, but increased CD11b protein expression (Fig. 1C). Ziftomenib treatment induced proteasomal degradation of Menin, since co-treatment with the proteasome inhibitor carfilzomib and ziftomenib restored Menin levels in MOLM13 cells (Supplementary Fig. S2C). Ziftomenib also increased MCL1 levels, unlikely due to MEK inhibition, as previously reported [10]. Genetic lesion(s) in TP53 were documented in approximately 9% of patients with MLL1-r AML [10]. Utilizing CRISPR/Cas9, the TP53 missense mutations R175H and R248Q, or null alleles, were introduced into the endogenous locus of MOLM13 cells that carry two wild-type copies of TP53, as previously reported [10, 12]. As compared to the control, MOLM13-TP53-R248Q cells harboring the missense mutation of TP53 (MOLM13-mtTP53 cells) were significantly less sensitive to the DNA-damaging drugs etoposide and ziftomenib (Fig. 1D) [10, 12]. Treatment with ziftomenib induced significantly more loss of viability in MOLM13-TP53-R175H and MOLM13-TP53^−/−^, as compared to MOLM13 cells (Fig. 1D).

Consistent with ziftomenib-mediated repression of mRNA and protein levels of BCL2, co-treatment with ziftomenib and venetoclax for 96 h exerted synergistic lethality, with delta synergy scores above 6.0 by the ZIP method (Supplementary Fig. S3A). This combination was also synergistically lethal against MV4-11 and OCI-AML3 cells. (Supplementary Figs. S3B and S3C). Consistent with the presence of FLT3-ITD mutation and expression, co-treatment with ziftomenib and the type I FLT3 kinase inhibitor gilteritinib exerted synergistic lethality in MOLM13 and MV4-11 cells (Supplementary Figs. S3D and S3E) [10]. BET protein BRD4 transcriptionally regulates enhancer-driven expressions of HOXA9-MEIS1 and their targets, as well as expressions of MYC, CDK4/6 and BCL2 [13]. Consistent with this, combined treatment with ziftomenib and OTX015 (a pan-BET protein inhibitor) yielded synergistic lethality in MV4-11, OCI-AML3 and MOLM13 cells (Supplementary Figs. S3F–S3H) [14]. Notably, this synergistic activity was also observed against MOLM13-TP53-R248Q cells (Supplementary Fig. S3I). Since ziftomenib treatment also inhibited protein levels of CDK6, we found that combined treatment with ziftomenib and abemaciclib (a CDK4/6 inhibitor) also caused synergistic loss of viability of MOLM13 and MV4-11 cells (Supplementary Figs. S3J and S3K) [10]. We also confirmed that IKAROS (encoded by IKZF1) is a dependency in KMT2A (MLL1)-rearranged (MLL1-r) and mtNPM1-expressing AML cells [15], by demonstrating that CRISPR knockout of IKZF1 significantly increased sensitivity of OCI-AML3 cells to ziftomenib (Supplementary Figs. S4A and S4B). Consistent with this, exposure to the IMID pomalidomide depleted IKZF1 and synergistically induced lethality with ziftomenib in OCI-AML3, MOLM13 and MV4-11 cells (Supplementary Figs. S4C–S4F).

**Fig. 2:**
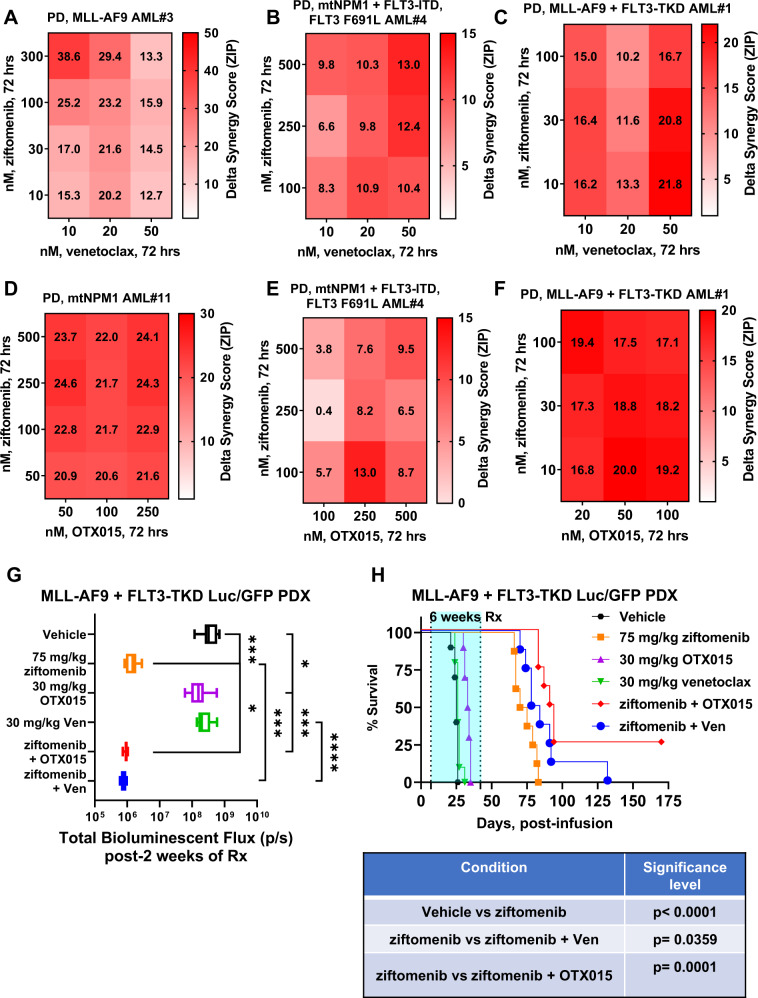
Co-treatment with ziftomenib and BCL2 inhibitor, venetoclax, or BET inhibitor OTX015 induces synergistic lethality in patient-derived AML cells with MLL1 rearrangement or mtNPM1 expression as well as significantly reduces in vivo leukemia burden and markedly improves median and overall survival of NSG mice bearing an MLL-AF9+FLT3-TKD-Luc/GFP AML PDX. **A**–**C** PD, MLL1-r and mtNPM1 expressing AML cells were treated with the indicated concentrations of ziftomenib and/or venetoclax for 72 h. At the end of treatment, cells were stained with To-Pro-3 iodide and the percentage of non-viable cells was determined by flow cytometry. Delta synergy scores for each combination were calculated utilizing the SynergyFinder V2 web application. **D**–**F** PD, MLL1-r and mtNPM1 expressing AML cells were treated with the indicated concentrations of ziftomenib and/or OTX015 for 72 h. At the end of treatment, cells were stained with To-Pro-3 iodide and the percentage of non-viable cells was determined by flow cytometry. Delta synergy scores for each combination were calculated utilizing the SynergyFinder V2 web application. **G** NSG mice were engrafted with luciferized patient-derived MLL-AF9+FLT3-TKD-expressing AML cells and monitored for 5–7 days. Mice were imaged by Xenogen camera, randomized to equivalent bioluminescence and treated with vehicle, 75 mg/kg of ziftomenib (PO, daily ×5 days) and/or 30 mg/kg of OTX015 (PO, daily ×5 days), or 30 mg/kg of venetoclax (PO, daily ×5 days) for 2 weeks. Total bioluminescent flux was determined for each cohort by Xenogen camera. *p < 0.05, ***p < 0.005, ****p < 0.001 as determined by a two-tailed, unpaired *t*-test. **H** Kaplan–Meier survival curve of NSG mice engrafted with luciferized patient-derived MLL-AF9+FLT3-TKD expressing AML cells and treated with ziftomenib and/or OTX015 or venetoclax for 6 weeks. Significant differences between cohorts were calculated with a log-rank test.

Utilizing mononuclear cells (containing >75% AML blasts) from 6 samples of bone marrow aspirate (BMA) or peripheral blood (PB) AML cells expressing MLL1-FP or mtNPM1 (oncoplot of co-mutations in Supplementary Fig. S5), we found that, treatment with ziftomenib (over control) significantly increased loss of viability, more so in AML expressing MLL1-FP than mtNPM1 (Fig. 1E). This was associated with a reduction in mRNA levels of MYC, PBX3, MEIS1 and BCL2 (Supplementary Figs. S6A and S6B). In both samples, ziftomenib treatment induced ITGAM expression (Supplementary Figs. S6A and S6B). Western blot analyses of cell lysates from one sample each of AML blasts with MLL1-r (sample #5) or with mtNPM1 (sample #11) demonstrated that treatment with ziftomenib reduced protein expressions of Menin, MEIS1, FLT3, CDK6, BCL2, with HOXA9 and MEF2C levels declining only in MLL1-r AML (Fig. 1F and Supplementary Fig. S6C). CyTOF analyses of three samples of phenotypically characterized AML stem/progenitor cells showed that ziftomenib treatment disparately reduced protein levels of Menin, HOXA9, MEIS1, PBX3, MEF2C, RUNX1, BCL2, CDK6, Ki67, Bcl-xL, MDM2 and BIM (Fig. 1G, H). Concomitantly, ziftomenib treatment caused a decline in the percentage of stem/progenitor cells in the three samples (Supplementary Figs. S6D and S6E). Co-treatment with ziftomenib and venetoclax also displayed synergistic lethal activity against PD AML cells harboring MLL-AF9 (sample #3), mtNPM1 (sample #11) and mtNPM1 plus FLT3-ITD and FLT3-TKD (sample #4) (Fig. 2A, B, and Supplementary Figs. S6F–S6H). Additionally, the combination of ziftomenib and OTX015 was also synergistically lethal against PD AML cells harboring either mtNPM1 (sample # 11) or mtNPM1 plus mtFLT3 (samples #2 and #4) (Fig. 2D, E, and Supplementary Figs. S6I–S6K). Notably, treatment with ziftomenib alone, or co-treatment of ziftomenib with venetoclax, or with lower doses of OTX015, either did not significantly or only modestly induce lethality in normal, cord blood derived, CD34+ progenitor cells (Supplementary Figs. S6L and S6M).

We next determined the in vivo anti-leukemia efficacy of ziftomenib and/or venetoclax or OTX015, at well-tolerated dose levels of these combinations, in NSG mice engrafted with PD AML cells harboring MLL1-r (MLL-AF9) and FLT3-TKD (sample #1). As shown in Fig. 2C, F and Supplementary Figs. S6N and S6O, these cells exhibited ex vivo synergistic lethality, following exposure to ziftomenib and venetoclax or OTX015. These cells had been transduced with and expressed Luciferase/GFP for bioluminescence imaging. Following tail-vein infusion and engraftment of these cells, cohorts of mice were treated with vehicle control, or with ziftomenib, venetoclax or OTX015 alone, or co-treatment with ziftomenib and venetoclax or OTX015. The dose of each drug employed here was relatively low and previously determined to be safe [10, 14]. Whereas monotherapy with relatively low dose of venetoclax or OTX015 alone for 2 weeks exhibited modest reduction of AML burden, treatment with ziftomenib alone induced marked and significant reduction in AML burden (Fig. 2G). Treatment with venetoclax or OTX015 alone failed to dramatically improve the overall survival of mice compared to those treated with vehicle control (Fig. 2G). In contrast, 6 weeks of treatment with ziftomenib alone yielded significant improvement in median and overall survival of the mice (*p* < 0.0001) (Fig. 2H). Notably, co-treatment with ziftomenib and venetoclax or OTX015 exhibited greater reduction of AML burden and significantly improved median and overall survival of the mice compared to treatment with vehicle or with each agent alone (Fig. 2G, H). Indeed, co-treatment with ziftomenib and OTX015 yielded a plateau in the survival curve up to 70 days (Fig. 2H). Neither monotherapy with the agents nor treatment with the combinations was associated with weight loss nor other toxicities, as compared to mice treated with vehicle alone.

In summary findings presented here highlight preclinically for the first time that ziftomenib treatment triggers Menin protein degradation through the ubiquitin-proteasome pathway and depletes Menin levels, as well as reduces protein expressions of MEF2C, MEIS1, FLT3, CDK6 and BCL2. This was associated with reversal of the differentiation block, induction of differentiation and loss of viability of AML cell lines and patient-derived AML cells harboring MLL1-r or mtNPM1 [8–10]. Our findings also identify that co-treatment of ziftomenib with BCL2, CDK6 or BET inhibitor induces synergistic in vitro lethality in AML cells with MLL1-r or mtNPM1. Furthermore, present findings also demonstrate that compared to MOLM13-TP53^+/+^ cells, ziftomenib drives more lethality in MOLM13-TP53-R175H and MOLM13-TP53^−/−^ cells. Finally, in a PDX model of AML cells harboring MLL-AF9 and FLT3 mutation, co-treatment with ziftomenib and venetoclax or OTX015 yielded significantly greater reduction in AML burden and improvement in survival compared to each agent alone without significant host toxicity. Overall, these findings support further in vivo testing and development of these MI-based combinations to not only improve the efficacy of ziftomenib, but also to undermine resistance to ziftomenib monotherapy in AML with MLL1-r or mtNPM1 that is encountered in the clinic.

## Supplementary information

Supplemental Data Figures

Supplemental Figure Legends

Supplemental Materials and Methods
