# Supplementary figures and images for: Activity of menin inhibitor ziftomenib (KO-539) as monotherapy or in combinations against AML cells with MLL1 rearrangement or mutant NPM1
Source: Leukemia. 2022 Sep 23;36(11):2729–33. doi: 10.1038/s41375-022-01707-w (PMC9613474; doi:10.1038/s41375-022-01707-w)

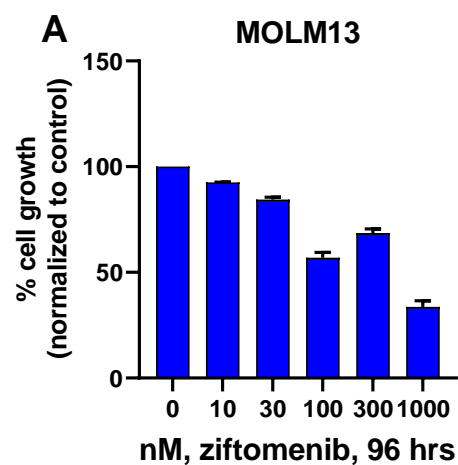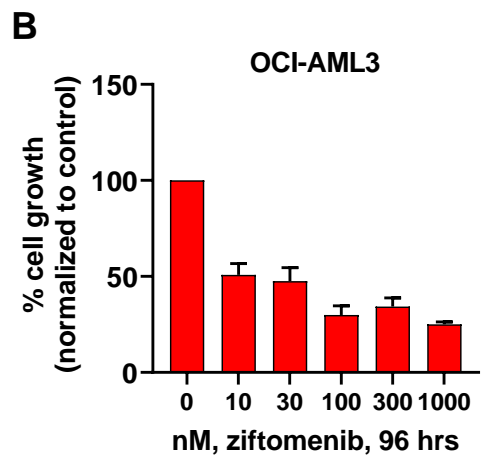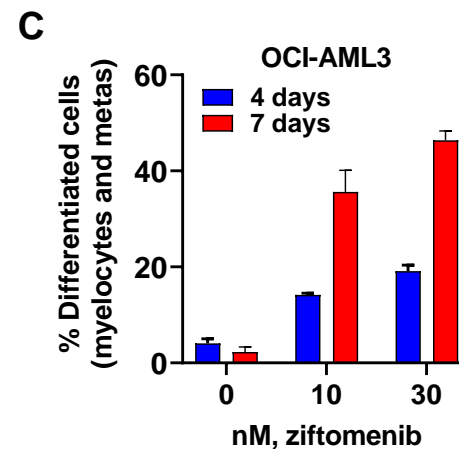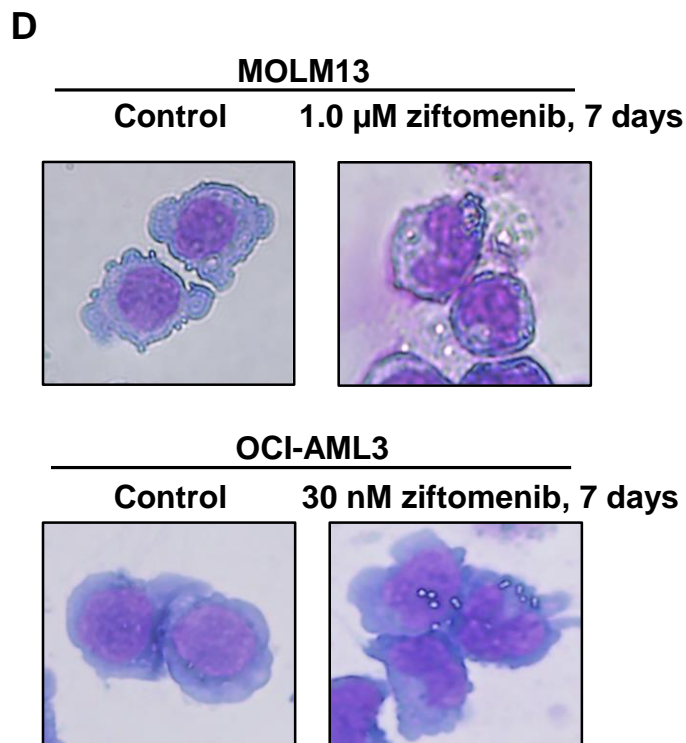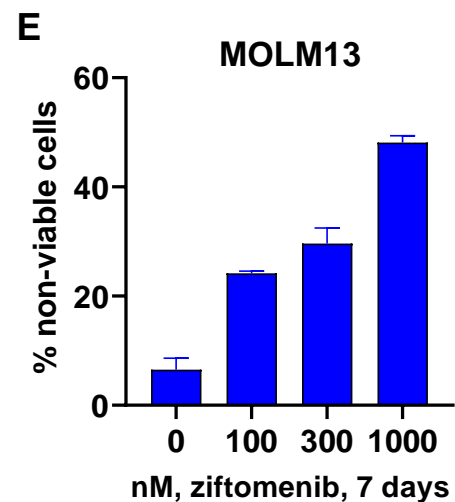

Figure S1

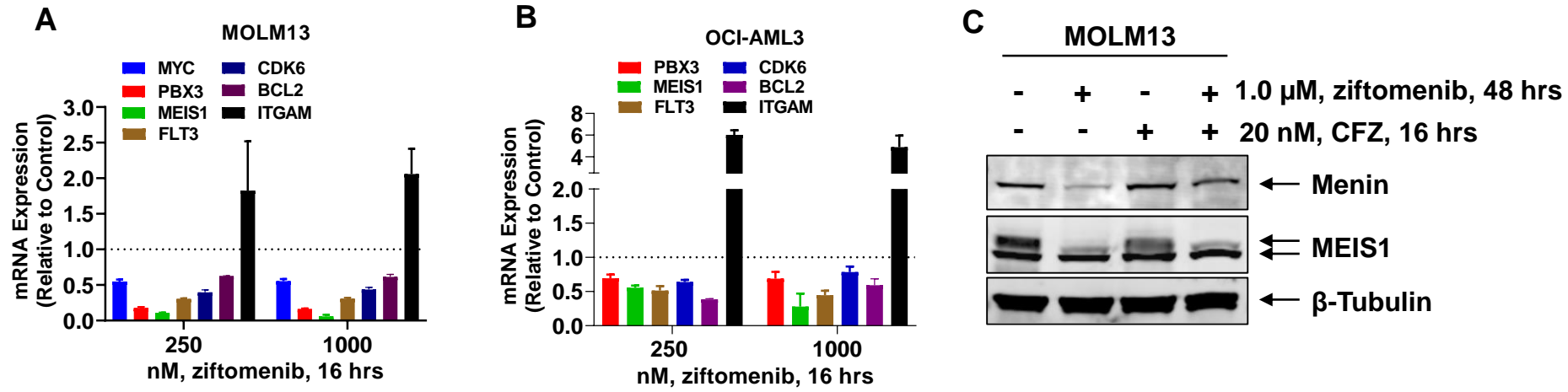

Figure S3

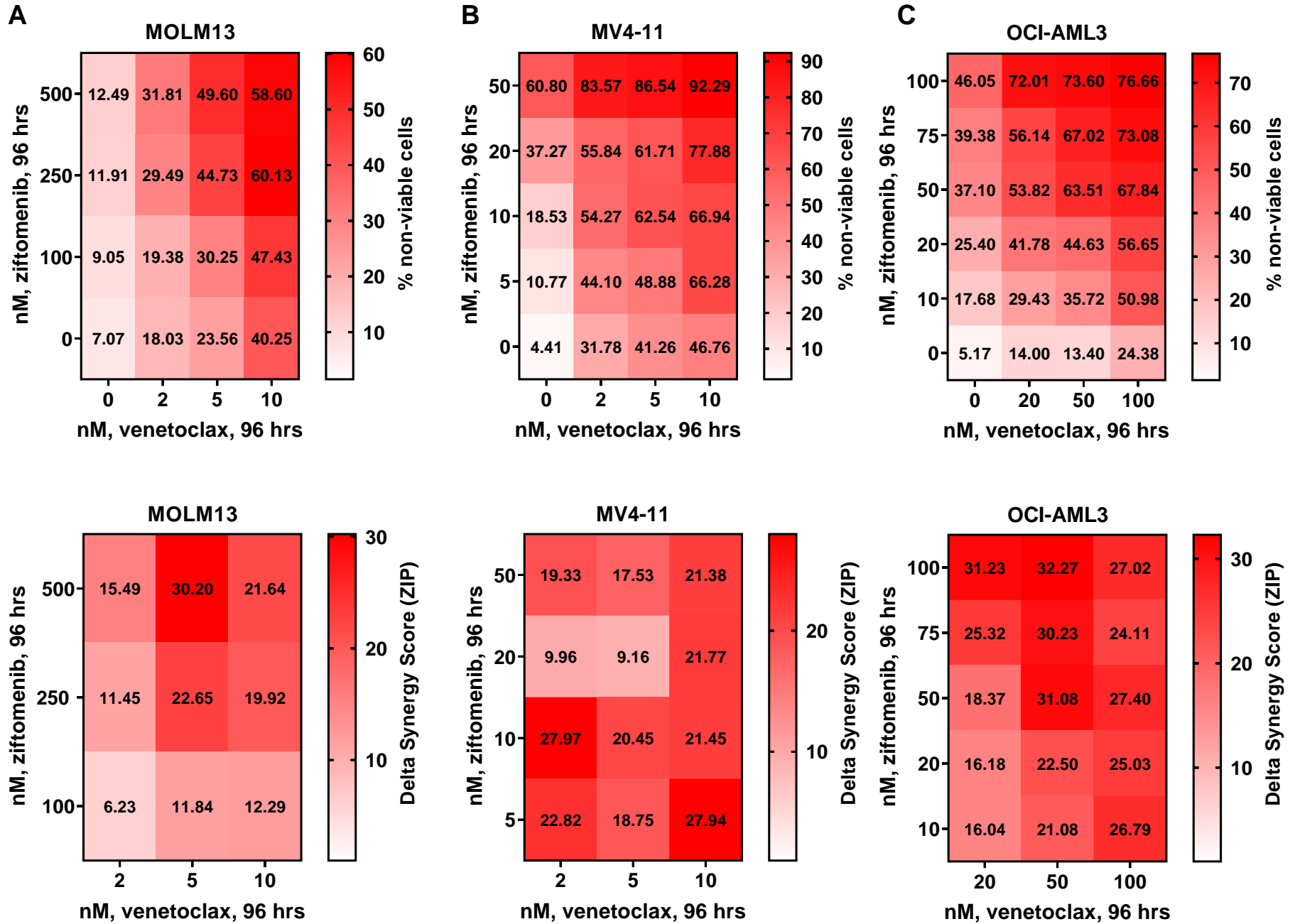

Figure S3

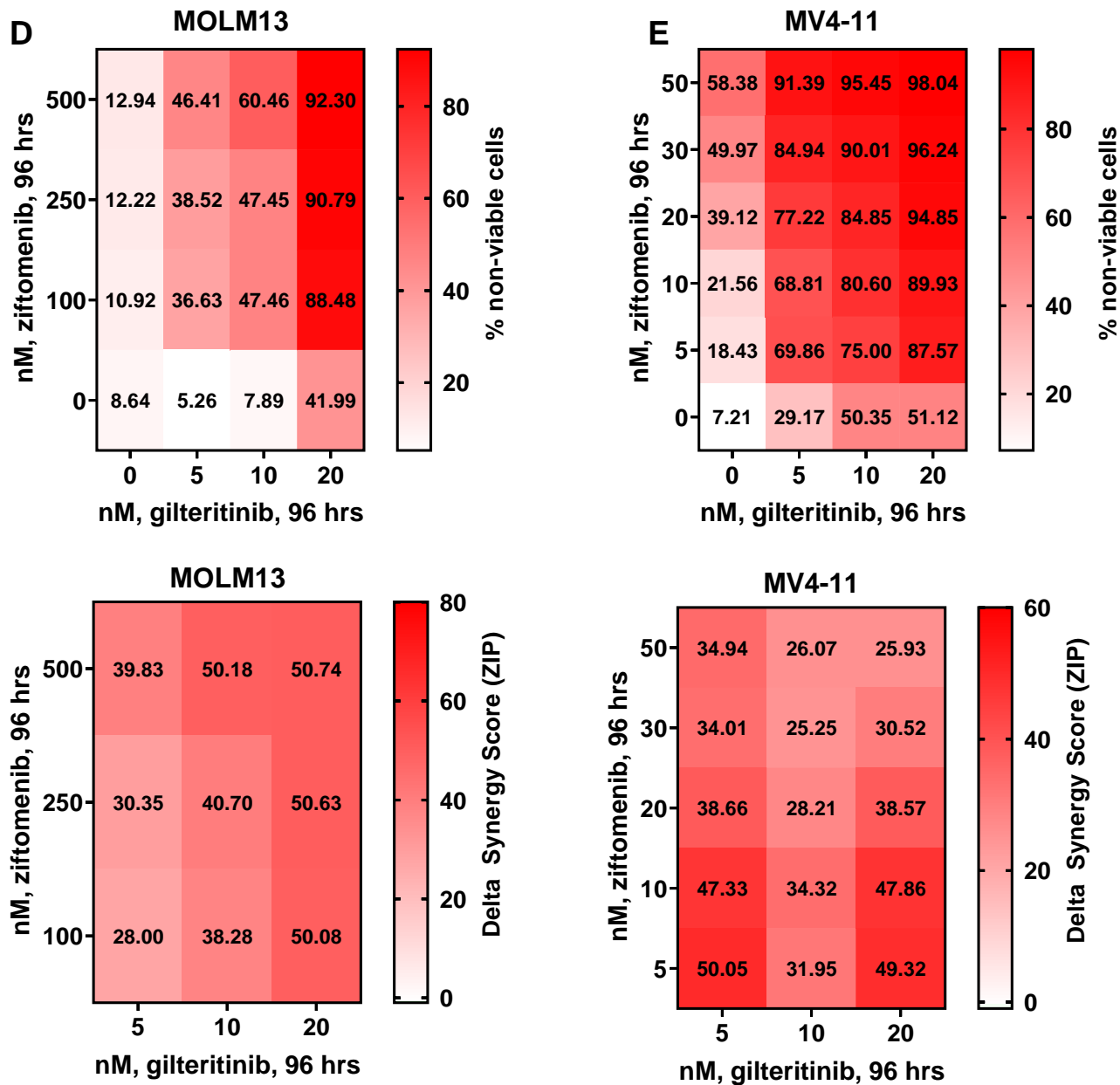

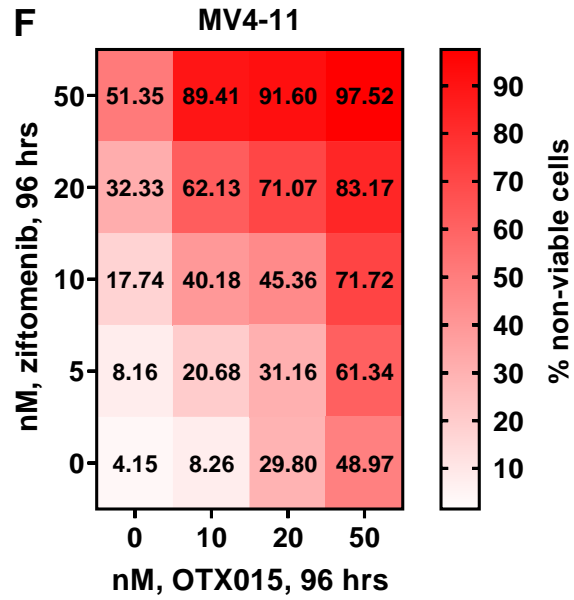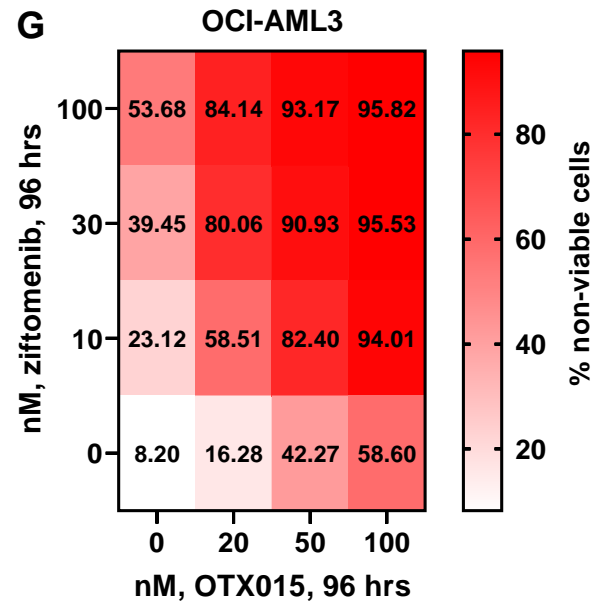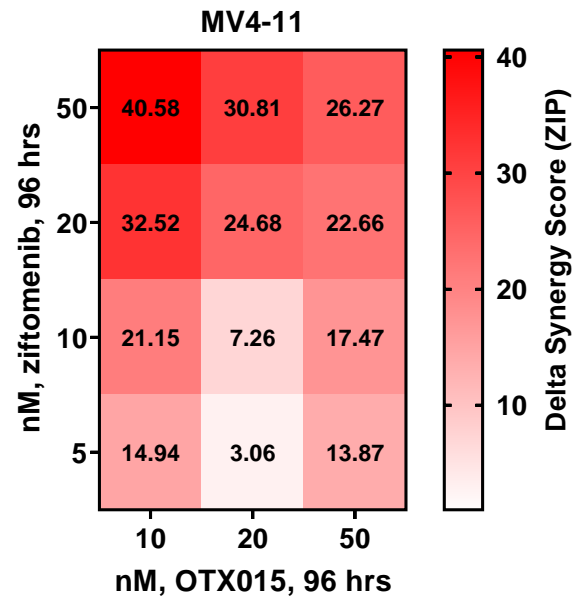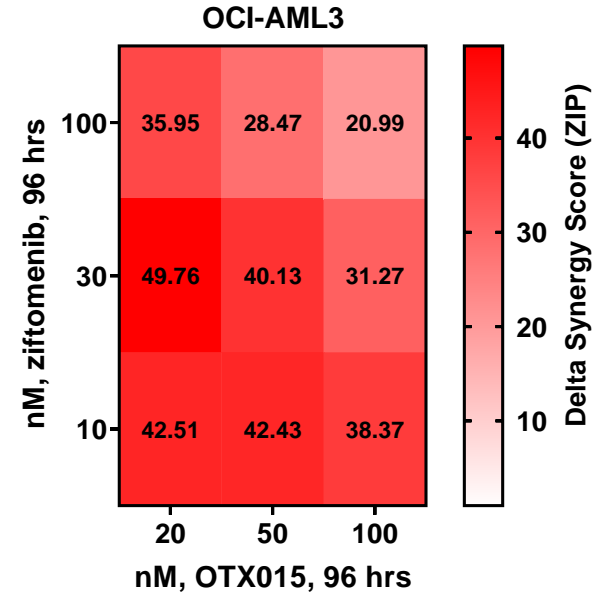

Figure S3

H

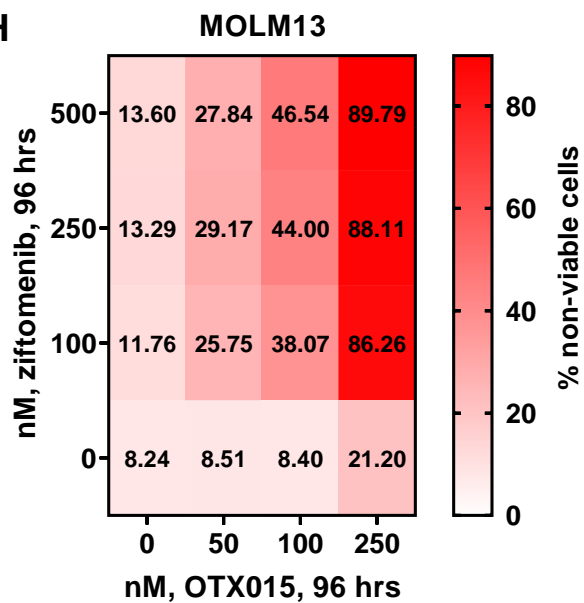

I

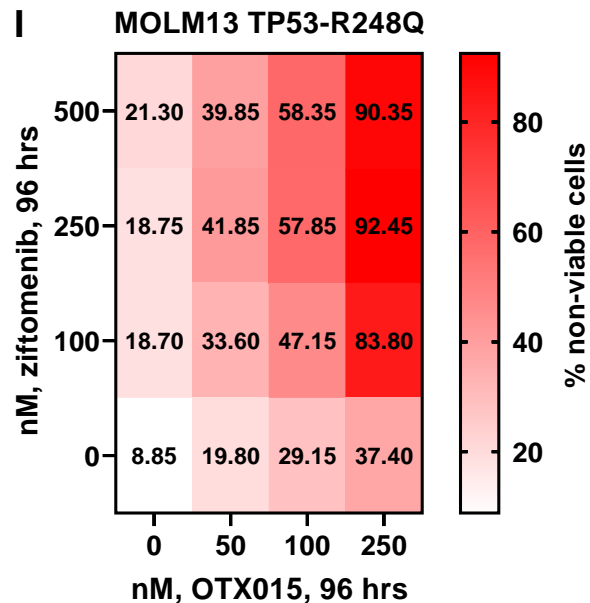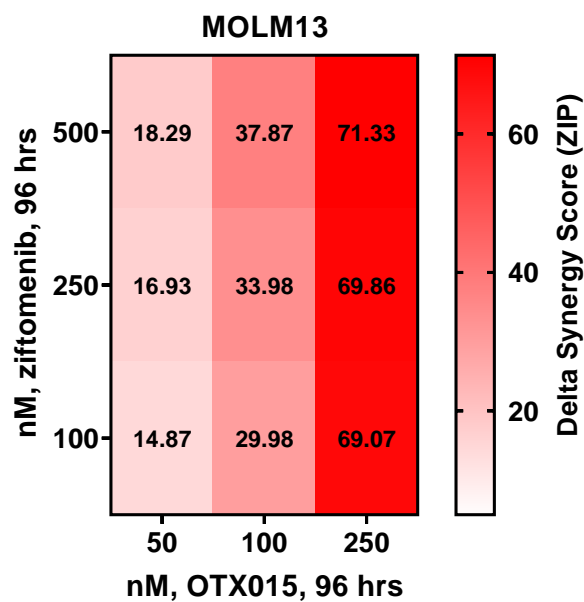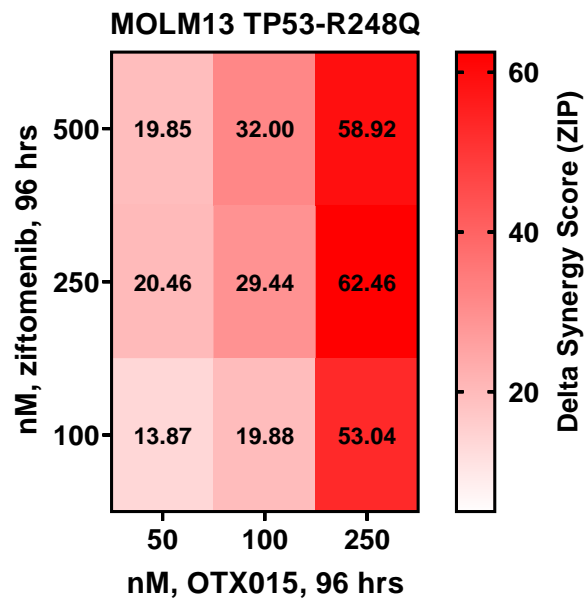

J

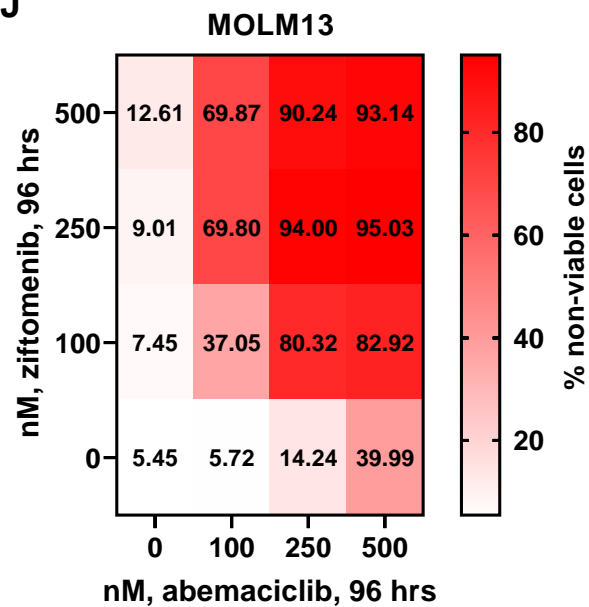

K

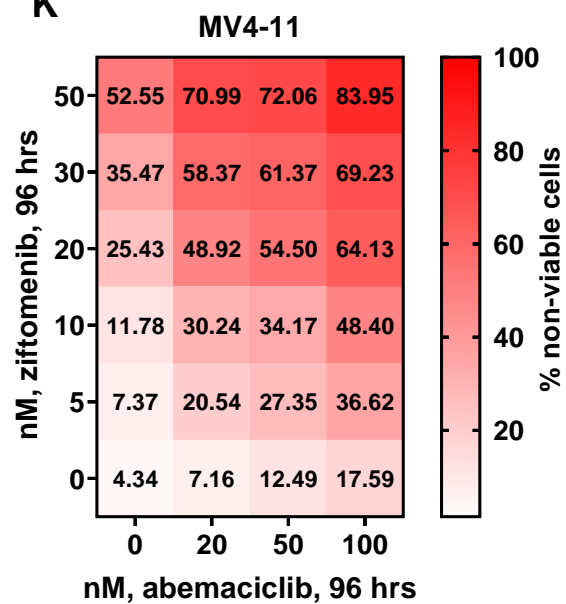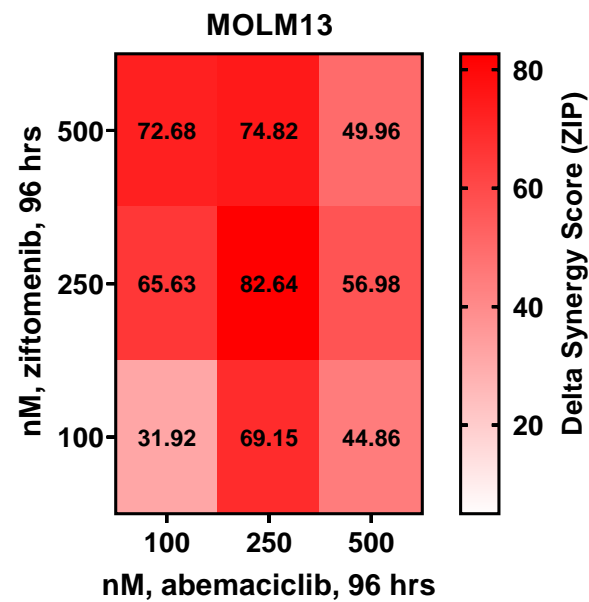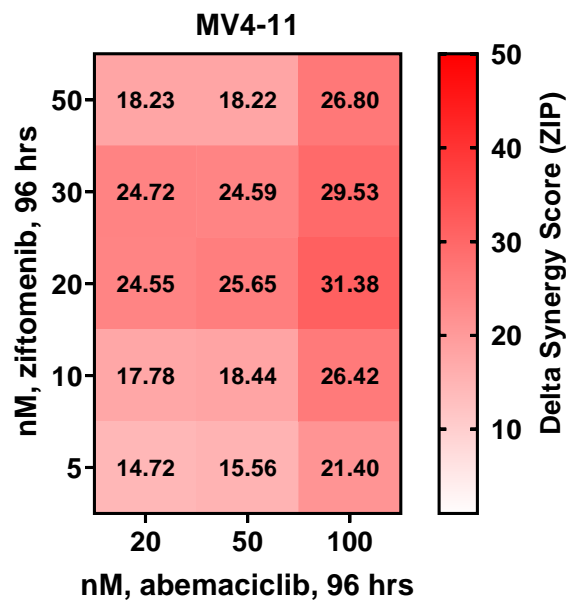

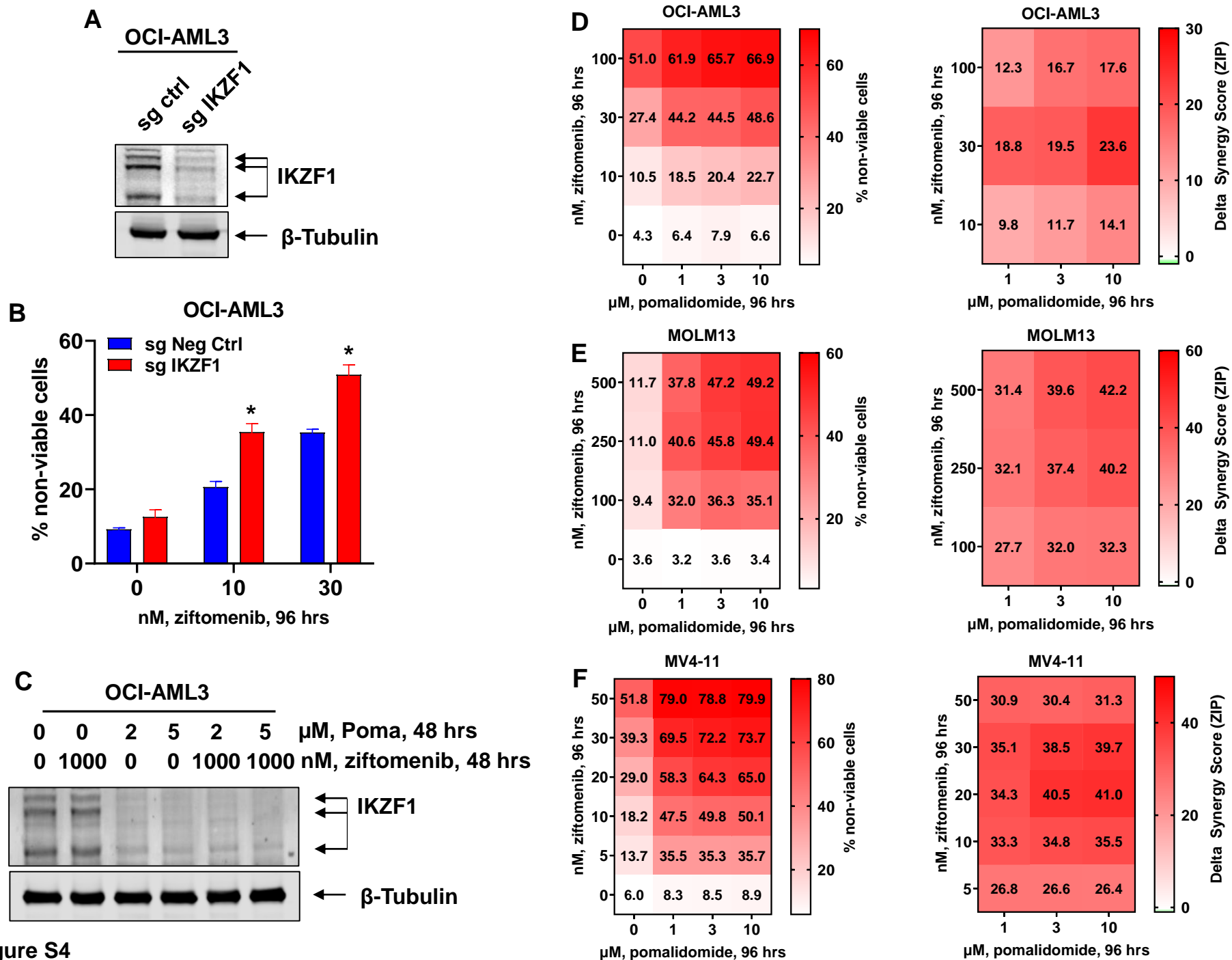

Figure S4



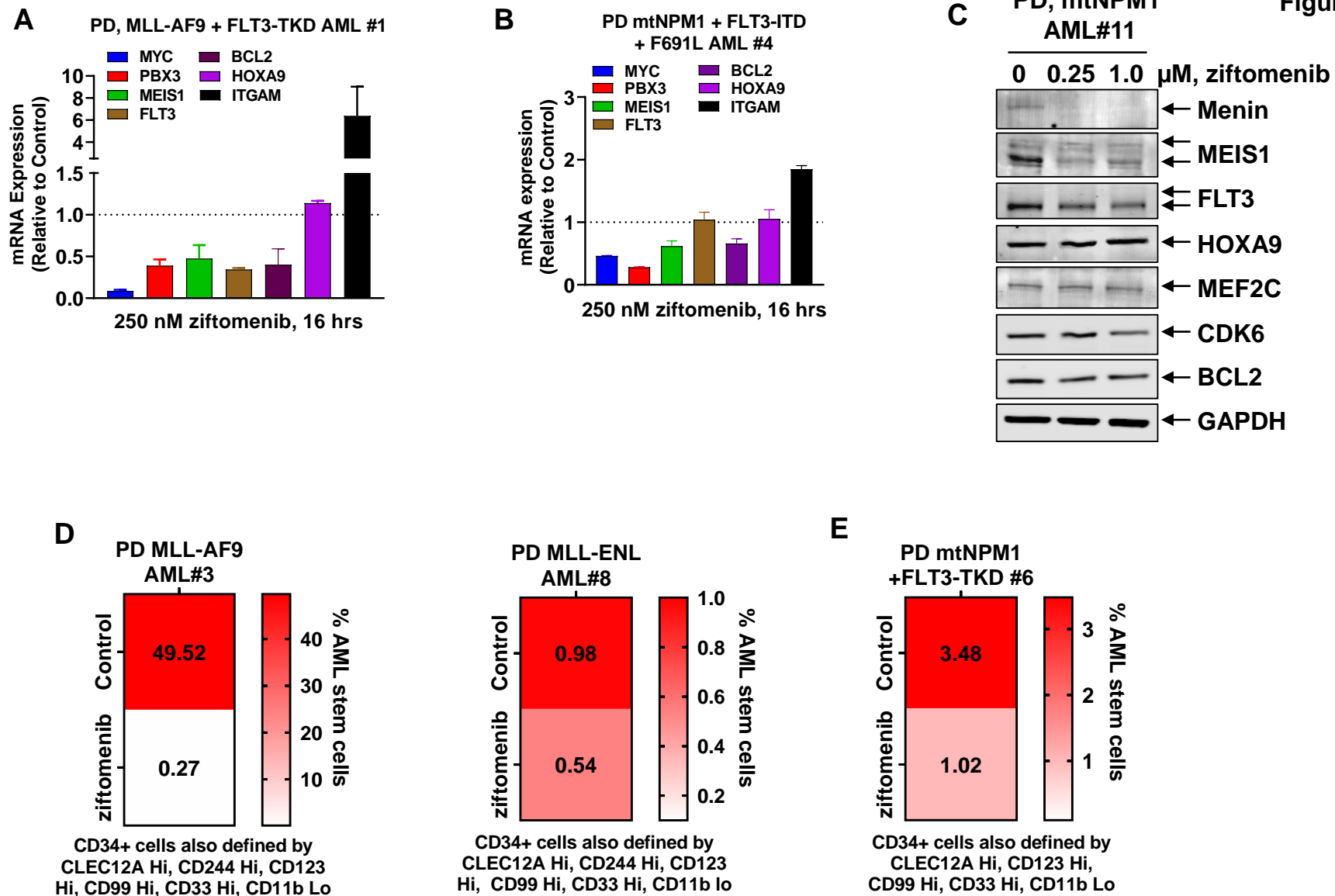

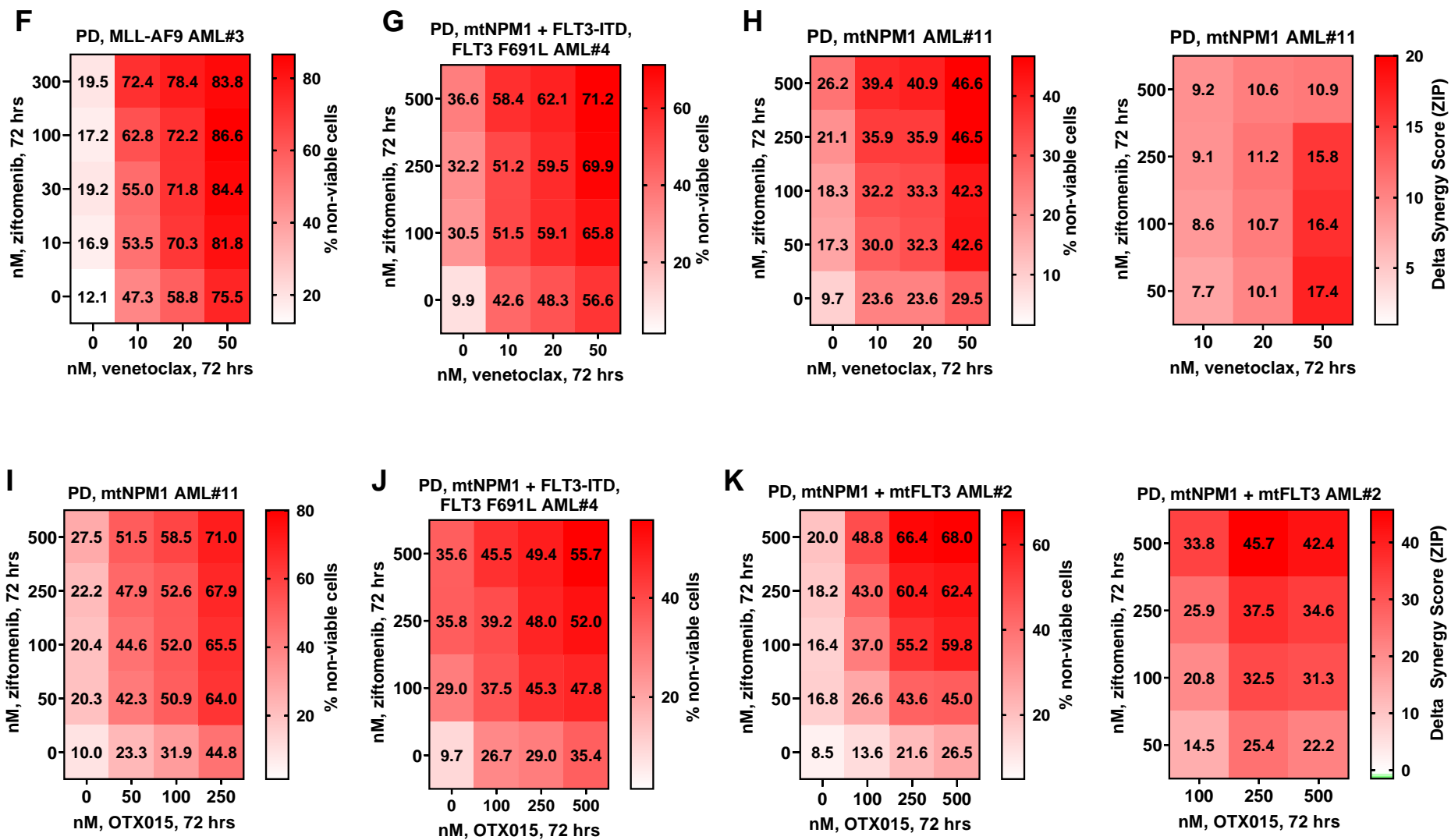

Figure S6

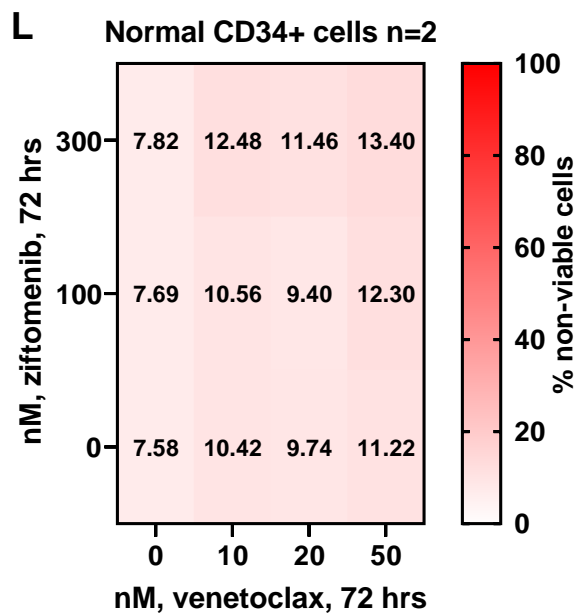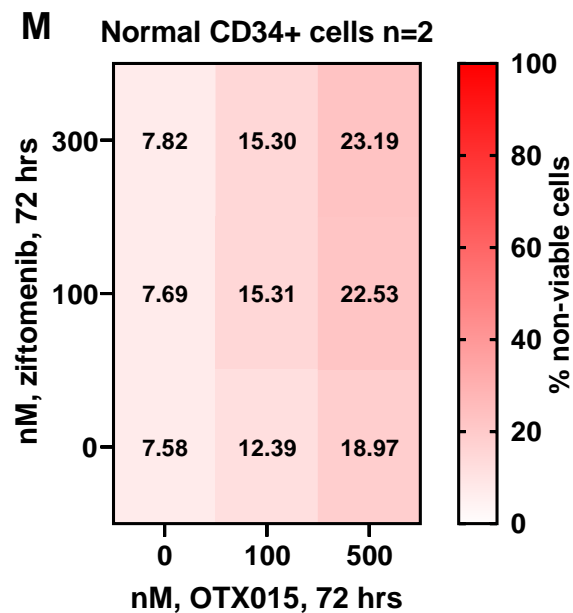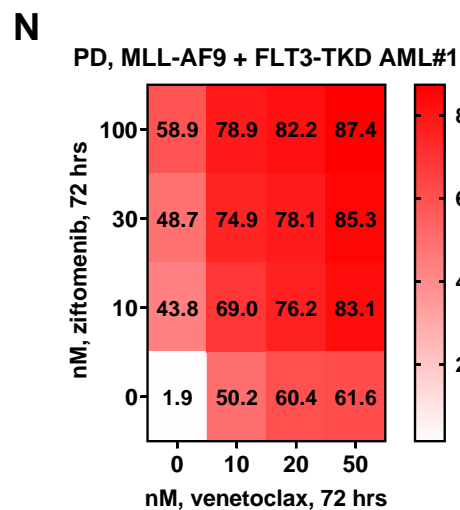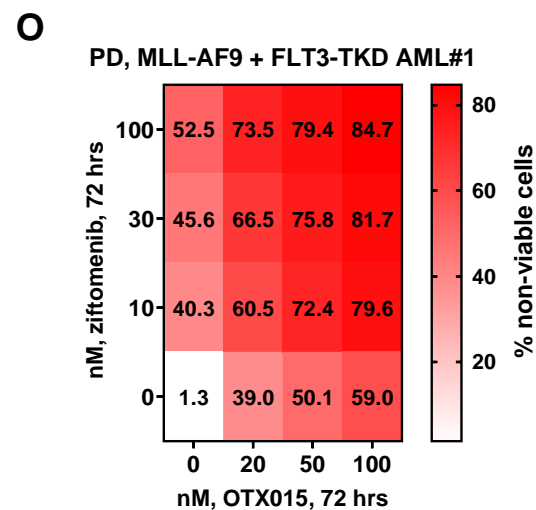

Supplement: Supplementary file 1 — Supplemental Data Figures [file 41375_2022_1707_MOESM1_ESM.pdf]
