# Supplementary material for: Activity of menin inhibitor ziftomenib (KO-539) as monotherapy or in combinations against AML cells with MLL1 rearrangement or mutant NPM1
Source: Leukemia. 2022 Sep 23;36(11):2729–33. doi: 10.1038/s41375-022-01707-w (PMC9613474; doi:10.1038/s41375-022-01707-w)
Supplement: Supplementary file 2 — Supplemental Figure Legends [file 41375_2022_1707_MOESM2_ESM.docx]

**Supplemental Figure Legends**

**Supplemental Figure 1. Treatment with Menin inhibitor ziftomenib induces growth inhibition and differentiation of AML cells with MLL1 rearrangement or mutant NPM1. A.** MOLM13 cells were treated with the indicated concentrations of ziftomenib for 96 hours. At the end of treatment, cell proliferation was determined using a Countess II cell counter. Columns, mean of three experiments + S.E.M. **B**. OCI-AML3 cells were treated with the indicated concentrations of ziftomenib for 96 hours. At the end of treatment, cell proliferation was determined using a Countess II cell counter. Columns, mean of three experiments + S.E.M. **C**. OCI-AML3 cells were treated with the indicated concentrations of ziftomenib for 4 and 7 days. Following this, features of morphologic differentiation were assessed in cells cytospun onto glass slides and stained with hematoxylin and eosin. Columns; mean of three experiments + S.E.M. **D**. MOLM13 and OCI-AML3 cells were treated with the indicated concentrations of ziftomenib for 7 days. Following this, cells were cytospun onto glass slides and stained with hematoxylin and eosin. Representative images (from two independent experiments) were obtained with a 40X objective and a CCD camera. **E.** MOLM13 cells were treated with the indicated concentrations of ziftomenib for 7 days. Following this, the % of To-Pro-3 iodide-positive, non-viable cells were determined by flow cytometry. Columns, mean of two independent experiments + S.D.

**Supplemental Figure 2. Treatment with ziftomenib alters mRNA expression of MLL1 target genes in MLL1-r mutant NPM1 expressing AML cells and co-treatment with proteasome inhibitor restores Menin expression levels.** **A-B**. MOLM13 and OCI-AML3 cells (biologic triplicates) were treated with the indicated concentrations of ziftomenib for 16 hours. Following this, total RNA was isolated, purified and reverse transcribed. The resulting cDNAs were utilized for qPCR analysis. In all cases, mRNA expression was normalized the expression of GAPDH and compared to the untreated control cells. **C**. Representative immunoblot analyses of MOLM13 cells treated with ziftomenib and/or proteasome inhibitor carfilzomib (CFZ) as indicated. The expression of β-Tubulin in the lysates served as the loading control.

**Supplemental Figure 3. Co-treatment with ziftomenib and venetoclax, gilteritinib, OTX015, or abemaciclib induces synergistic lethality in AML cells expressing mtNPM1 or MLL1 rearrangement with or without mutant TP53 expression.** **A-C**. MOLM13, MV4-11, and OCI-AML3 cells were treated with the indicated concentrations of ziftomenib and/or BCL2 inhibitor venetoclax for 96 hours. At the end of treatment, cells were stained with To-Pro-3 iodide and the percentage of non-viable cells was determined by flow cytometry. Delta synergy scores for each combination (from two independent experiments) were calculated utilizing the SynergyFinder V2 web application. **D-E**. MOLM13 and MV4-11 cells were treated with the indicated concentrations of ziftomenib and/or FLT3 inhibitor gilteritinib for 96 hours. At the end of treatment, cells were stained with To-Pro-3 iodide and the percentage of non-viable cells was determined by flow cytometry. Delta synergy scores for each combination (from two independent experiments) were calculated utilizing the SynergyFinder V2 web application. **F-I**. MV4-11, OCI-AML3, MOLM13, and MOLM13 TP53-R248Q cells were treated with the indicated concentrations of ziftomenib and/or pan-BET inhibitor OTX015 for 96 hours. At the end of treatment, cells were stained with To-Pro-3 iodide and the percentage of non-viable cells was determined by flow cytometry. Delta synergy scores for each combination (from two independent experiments) were calculated utilizing the SynergyFinder V2 web application. **J-K**. MOLM13 and MV4-11 cells were treated with the indicated concentrations of ziftomenib and/or CDK4/6 inhibitor abemaciclib for 96 hours. At the end of treatment, cells were stained with To-Pro-3 iodide and the percentage of non-viable cells was determined by flow cytometry. Delta synergy scores for each combination (from two independent experiments) were calculated utilizing the SynergyFinder V2 web application.

**Supplemental Figure 4. Knockout of IKZF1 enhances sensitivity of AML cells to Menin inhibitor treatment and co-treatment with ziftomenib and the immunomodulatory imide (IMiD) pomalidomide induces synergistic lethality in MLL1-r and mtNPM1-expressing AML cells. A**. OCI-AML3 cells were transfected with sg-Neg control guide or sg-IKZF1 and incubated for 5 days. Immunoblot analyses were conducted on total cell lysates. Representative immunoblots are shown for IKZF1. **B**. OCI-AML3 cells were transfected with sg-Neg control guide or sg-IKZF1 and incubated for 3 days. Then, cells were treated with the indicated concentrations of ziftomenib for 96 hours and the % non-viable cells was determined by To-Pro-3 iodide staining and flow cytometry. Columns; mean of two independent experiments + S.D. **C.** OCI-AML3 cells were treated with the indicated concentrations of ziftomenib and/or pomalidomide (Poma) for 48 hours. At the end of treatment, total cell lysates were prepared and immunoblot analyses were conducted. The expression of β-Tubulin in the cell lysates served as the loading control. A representative immunoblot from two independent experiments is shown. **D-F**. OCI-AML3, MOLM13, and MV4-11 cells were treated with the indicated concentrations of ziftomenib and/or pomalidomide for 96 hours. At the end of treatment, cells were stained with To-Pro-3 iodide and the percentage of non-viable cells was determined by flow cytometry. Delta synergy scores for each combination were calculated from two independent experiments utilizing the SynergyFinder V2 web application.

**Supplemental Figure 5. Oncoplot of the mutations identified (by Next-Gen sequencing of an 81-gene panel) in the patient-derived (PD), de novo AML samples utilized in these studies.** The number of each patient corresponds to the patient-derived AML samples shown in Figure 1, Figure 2 and Figure S6.

**Supplemental Figure 6. Treatment with ziftomenib depletes leukemia stem/progenitor cells and co-treatment with ziftomenib and venetoclax, or BET inhibitor OTX015 induces synergistic lethality in PD AML cells with MLL1 rearrangement or expressing mtNPM1 but exhibits minimal lethal activity against CD34+ normal hematopoietic progenitor cells (HPCs). A-B**. PD, MLL1-r and mtNPM1 expressing AML cells were treated in duplicates with the indicated concentration of ziftomenib for 16 hours. Following this, total RNA was isolated, purified and reverse transcribed. The resulting cDNAs were utilized for qPCR analysis. In all cases, mRNA expression was normalized the expression of GAPDH and compared to the untreated control cells.  **C.** Patient-derived, mtNPM1-expressing AML cells were treated with the indicated concentrations of ziftomenib for 48 hours. Total cell lysates were prepared and immunoblot analyses were conducted. The expression levels of GAPDH in the cell lysates served as the loading control. **D-E**. PD, MLL1-rearranged and mtNPM1-expressing AML cells were treated with 1 µM of ziftomenib for 16 hours. Then, cells were incubated with cocktails of rare, heavy metal ion-tagged antibodies against extracellular and intracellular proteins. Mass cytometry (CyTOF) analysis was performed on the untreated and treated cells and the data were analyzed by Astrolabe. Panels show the percentage of phenotypically defined AML stem cells present in the PD AML cells before and after treatment with ziftomenib. **F-H**. PD, MLL1-r and mtNPM1 expressing AML cells were treated with the indicated concentrations of ziftomenib and/or venetoclax for 72 hours. At the end of treatment, cells were stained with To-Pro-3 iodide and the percentage of non-viable cells was determined by flow cytometry. Delta synergy scores for each combination were calculated utilizing the SynergyFinder V2 web application. **I-K**. PD, MLL1-r and mtNPM1 expressing AML cells were treated with the indicated concentrations of ziftomenib and/or OTX015 for 72 hours. At the end of treatment, cells were stained with To-Pro-3 iodide and the percentage of non-viable cells was determined by flow cytometry. Delta synergy scores for each combination were calculated utilizing the SynergyFinder V2 web application. **L-M**. Normal CD34+ HPCs from cord blood (n=2) were treated with the indicated concentrations of ziftomenib and venetoclax or OTX015 for 72 hours. Following this, cells were stained with To-Pro-3 iodide and the percentage of non-viable cells was determined by flow cytometry. **N-O**. PD, MLL1-r and FLT3-TKD expressing AML cells were treated with the indicated concentrations of ziftomenib and/or venetoclax or OTX015 for 72 hours. At the end of treatment, cells were stained with To-Pro-3 iodide and the percentage of non-viable cells was determined by flow cytometry.
