# Supplementary material for: Activity of menin inhibitor ziftomenib (KO-539) as monotherapy or in combinations against AML cells with MLL1 rearrangement or mutant NPM1
Source: Leukemia. 2022 Sep 23;36(11):2729–33. doi: 10.1038/s41375-022-01707-w (PMC9613474; doi:10.1038/s41375-022-01707-w)
Supplement: Supplementary file 3 — Supplemental Materials and Methods [file 41375_2022_1707_MOESM3_ESM.docx]

**Supplemental Materials and Methods:**

**Contact for Reagent sharing. Kapil N. Bhalla. Department of Leukemia, MD. Anderson Cancer Center, 1400 Holcombe Blvd, Unit428, Houston, TX, 77030. kbhalla@mdanderson.org**

**Reagents and antibodies.** Venetoclax, OTX015, gilteritinib, pomalidomide, carfilzomib, and abemaciclib were obtained from MedChem Express (Monmouth Junction, NJ). Ziftomenib (KO-539) for in vitro and in vivo studies was obtained from Kura Oncology (San Diego, CA) under a material transfer agreement. All compounds were prepared as 10 mM stocks in 100% DMSO and frozen at -80°C in 5-10 µL aliquots to allow for single use, thus avoiding multiple freeze-thaw cycles that could result in compound decomposition and loss of activity. Anti-c-Myc [RRID:AB_1903938], anti-MCL1 [RRID:AB_2799149], anti-Bcl-xL [RRID:AB_10695729], anti-Menin [RRID:AB_10858216], anti-β-Tubulin [RRID:AB_2715541], anti-MEF2C [RRID:AB_10548759], and IKZF1 (Ikaros, clone D10E5) [RRID:AB_2797691] antibodies were obtained from Cell Signaling Technologies (Beverly, MA). Anti-MEIS1 [RRID:AB_776272], anti-FLT3 [ab245116], anti-HOXA9 [ab140631], anti-PBX3 [RRID:AB_10858991], and anti-CD11b [RRID:AB_2650514] antibodies were obtained from Abcam (Cambridge, MA). Anti-BFL1 [# ABC490] antibody was obtained from Millipore/Sigma (Burlington, MA). Anti-CDK6 [RRID:AB_10610066], anti-BCL2 [RRID:AB_626733], and anti-GAPDH [RRID:AB_627679] antibodies were obtained from Santa Cruz Biotechnologies (Santa Cruz, CA). Anti-p27 [RRID:AB_397636] antibody was obtained from BD Transduction Labs (Franklin Lakes, NJ).

**Cell lines and cell culture.** MOLM13 [DSMZ Cat# ACC-554, RRID: CVCL_2119], OCI-AML3 [DSMZ Cat# ACC-582, RRID:CVCL_1844] and NOMO-1 [DSMZ Cat# ACC-542 RRID: CVCL_1609] cells were obtained from the DSMZ. MV4-11 [ATCC Cat# CRL-9591, RRID:CVCL_0064] and THP1 [RRID:CVCL_0006] cells were obtained from the ATCC (Manassas, VA). MOLM13 cells with isogenic TP53 mutations [R175H, R248Q and TP53-KO] were a gift from Dr. Benjamin L. Ebert (Dana Farber Cancer Center, Boston, MA). All experiments with cell lines were performed within 6 months after thawing or obtaining from ATCC or DSMZ. The cell lines were also authenticated in the Characterized Cell Line Core Facility at M.D. Anderson Cancer Center, Houston TX. MOLM13, NOMO-1, THP1, and OCI-AML3 cells were cultured in RPMI-1640 media with 20% FBS and 1% penicillin/streptomycin. MV4-11 cells were cultured in ATCC-formulated IMDM media with 20% FBS and 1% penicillin/streptomycin. Logarithmically growing, mycoplasma-negative cells were utilized for all experiments. Following drug treatments, cells were washed free of the drug(s) prior to the performance of the studies described.

**Cell Line Authentication**. The cell lines utilized in these studies were authenticated in the Characterized Cell Line Core Facility at M.D. Anderson Cancer Center, Houston TX utilizing STR profiling.

**Primary AML blasts:** Patient-derived AML cells samples were obtained with informed consent as part of a clinical protocol approved by the Institutional Review Board of The University of Texas, M.D. Anderson Cancer Center. Mononuclear cells were purified by Ficoll Hypaque (Axis Shield, Oslo, Norway) density centrifugation following the manufacturer’s protocol. Mononuclear cells were washed once with sterile 1X PBS then suspended in complete RPMI media containing 20% FBS. Cells were counted to determine the number of cells isolated prior to immuno-magnetic selection. CD34+ AML blast progenitor cells were purified by immuno-magnetic beads conjugated with anti-CD34 antibody following the manufacturer’s protocol (StemCell Technologies, Vancouver, British Columbia) prior to utilization in the cell viability assays, RNA expression, and immunoblot analyses.

**Sequencing of primary de novo blast cells:** We performed targeted next-generation sequencing (NGS) of DNA samples from bone marrow or peripheral blood collected from patients at our center with de novo AML (1). Diagnostic bone marrow samples were obtained for mutational analysis. Total genomic DNA was extracted from unenriched peripheral blood (PB) or bone marrow (BM) samples using ReliaPrep genomic DNA isolation kit (Promega Corp, Madison, WI, USA). Briefly, a total of 250 ng DNA was utilized to prepare sequencing libraries using Agilent HaloPlex custom Kit (Agilent Technologies, Santa Clara, CA, USA). The entire coding sequences of 81 genes including ABL1, ASXL1, BRAF, CALR, DNMT3A, EGFR, EZH2, FLT3, GATA1, GATA2, HRAS, IDH1, IDH2, KIT, KRAS, MDM2, IKZF2, JAK1, JAK2, MLL, MPL, MYD88, NOTCH1, NF1, NPM1, NRAS, PTPN11, RUNX1, TET2, TP53, and WT1 were interrogated on a custom-designed next-generation sequencing approach using the Illumina MiSeq platform [Illumina; San Diego, CA, USA; RRID:SCR_016379]. The genomic reference sequence used was genome GRch37/hg19. The following software tools were utilized in the experimental setup and data analysis: Illumina Experiment Manager 1.6.0 (Illumina; San Diego, CA, USA), MiSeq Control Software 2.4 (Illumina; San Diego, CA, USA), Real Time Analysis 1.18.54 (Illumina; San Diego, CA, USA), Sequence Analysis Viewer 1.8.37 (Illumina; San Diego, CA, USA), MiSeq Reporter 2.5.1 (Illumina; San Diego, CA, USA), and SureCall 3.0.1.4 (Agilent Technologies; Santa Clara, CA, USA). A minimum of 80% reads at quality scores of AQ30 or higher were required to pass quality control. The lower limit of detection of this assay (analytical sensitivity) for single nucleotide variations was determined to be 5% (one mutant allele in the background of nineteen wild type alleles) to 10% (one mutant allele in the background of nine wild type alleles). Testing of patients with active hematologic malignancies was limited to somatic mutations only.

**CRISPR/Cas9-mediated gene editing in cultured AML cells.** To study the effects of knockout of IKZF1 in AML cells, the CHOP-CHOP prediction algorithm (2) was utilized to develop guide RNAs. High scoring sgRNAs were synthesized by Synthego, Inc. For OCI-AML3 cells without stable Cas9 expression, to obtain Cas9-sgRNA RNPs (ribonucleoprotein complexes), 1 μg of synthetic sgRNA was incubated with 1.5 μg recombinant Cas9 protein (Synthego, Inc.) for 15 min at room temperature. OCI-AML3 cells were transfected by electroporation utilizing the Amaxa 2D-Nucleofector device with a Cell Line-Specific Nucleofector Kit (Amaxa GmbH, Cologne, Germany) as per the manufacturer's instructions and program X-001. Immediately post-transfection, cells were plated in complete media containing no antibiotics and 2 µM of ROCK inhibitor (Y-27632, Selleck Chemicals; Houston, TX) and allowed to recover for 24 hours. Knockout of IKZF1 was confirmed by Western blot analysis 5 days post-transfection. Gene-edited AML cells were treated with Menin inhibitor for 96 hours and the % of To-Pro-3 iodide-positive, non-viable cells was determined by flow cytometry.

**Assessment of percentage non-viable cells.** Following designated treatments (72-96 hours), cultured cell lines or PD- AML cells, were washed with 1X PBS, stained with TO-PRO-3 iodide (Life Technologies, Carlsbad, CA) and analyzed by flow cytometry on a BD Accuri CFlow-6 flow cytometer (BD Biosciences, San Jose, CA). We used matrix dosing of agents in combinations to allow synergy assessment utilizing the SynergyFinder V2 online web application tool (<http://synergyfinder.fimm.fi/>). Delta synergy scores were generated utilizing the ZIP method (3, 4).

**Assessment of leukemia cell differentiation.** Following treatment with Menin inhibitors, cells were harvested and washed with 1X PBS. Cells were re-suspended in 0.5% BSA/PBS and stained with APC-conjugated anti-CD11b antibody [RRID:AB_398456] or APC-conjugated IgG1 isotype control antibody [RRID:AB_398613] in the dark, at 4°C for 15-20 minutes. Cells were washed with 0.5% BSA/PBS by centrifugation at 125 x g for 5 minutes, and then suspended in 0.5% BSA/PBS for analysis by flow cytometry. Cells were assessed in the FL-4 fluorescence channel on a BD Accuri CFLow6 flow cytometer. Differentiation of leukemia cells was also determined by examination of cellular/nuclear morphology. Cells were cytospun onto glass slides at 500 rpm for 5 minutes. The cytospun cells were fixed and stained with a Protocol® HEMA3 stain set (Fisher Scientific, Kalamazoo, MI). Cellular/nuclear morphology was assessed by light microscopy. Two hundred cells were counted in at least 5 different sections of the slide for each condition. The % morphologic differentiation is reported relative to the control cells. Each experiment was performed at least twice.

**RNA isolation and quantitative polymerase chain reaction.** Following the designated treatments, total RNA was isolated from cultured or patient-derived AML cells utilizing a PureLink RNA Mini kit from Ambion, Inc. (Austin, TX) and reverse transcribed with a High Capacity Reverse Transcription kit from Life Technologies (Carlsbad, CA). Quantitative real-time PCR analysis for the expression of target genes was performed on cDNA using TaqMan probes and a TaqMan Universal PCR Mastermix from Applied Biosystems (Foster City, CA). Relative mRNA expression was normalized to the expression of GAPDH and compared to the untreated cells.

**Cell lysis and protein quantitation.** Untreated or drug-treated cells were centrifuged, and the cell pellets were incubated in lysis buffer on ice for 20 minutes (5). After centrifugation, an aliquot of each cell lysate was diluted 1:10 and the protein content was quantitated using a BCA protein quantitation kit (Pierce, Rockford, IL), according to the manufacturer’s protocol. Protein concentrations were determined by comparing the absorbance at 562 nm compared to a known concentration range of bovine serum albumin (BSA) from 0.125 mg/mL to 2 mg/mL.

**SDS-PAGE and immunoblot analyses.** Seventy-five micrograms of total cell lysate were used for SDS-PAGE. Western blot analyses were performed on total cell lysates using specific antisera or monoclonal antibodies. Blots were washed with 1× PBST, then incubated in IRDye 680RD goat anti-mouse (RRID:AB_10956588) or IRDye 800CW goat anti-rabbit (RRID:AB_621843) secondary antibodies (LI-COR, Lincoln, NE) for 1 h, washed three times in 1× Phosphate Buffered Saline with Tween®20 (PBST) and scanned with an Odyssey CLX Infrared Imaging System utilizing Image Studio 5.0 Software (RRID:SCR_015795) (LI-COR, Lincoln, NE). The expression levels of β-Actin or GAPDH in the cell lysates were used as the loading control for the western blots. Immunoblot analyses were performed at least twice. Representative immunoblots are shown

**Single cell next-generation mass cytometry ‘CyTOF’ analysis of PD MLL1-r or mtNPM1 expressing AML cells.** Primary, patient-derived MLL1r or mtNPM1 expressing AML cells were treated with 1.0 µM of ziftomenib for 16 hours. At the end of treatment, cells were blocked with staining buffer (0.5% BSA/PBS) for 30 minutes, then a cocktail of extracellular antibodies conjugated to transition element isotopes were added and incubated for 1 hour at room temperature (RT). For viability staining, 5 µM Cisplatin was added and incubated at RT for 2 minutes. Cells were washed with staining buffer, centrifuged at 500 x g for 5 minutes and staining buffer was vacuum aspirated. Cells were fixed with 100 µL of 1.6% paraformaldehyde (PFA) for 10 minutes at room temperature. Following this, cells were permeabilized with 900 µL of ice-cold 100% methanol (90% volume) at -20°C for at least 20 minutes. Next, cells were washed with 1 ml of staining buffer to remove the paraformaldehyde/methanol solution. Cells were blocked in 50 µL of staining buffer for 30 minutes and a cocktail of intracellular antibodies conjugated to transition element isotopes was added to be used as tags in atomic mass spectrometric analysis of the cells. Cells were incubated for 1 hour at room temperature, then washed with staining buffer at 500 x g for 5 minutes. Intercalator was added (500 µL of 1:1000 Ir-intercalator diluted in 1.6% PFA/1X PBS) and cells were incubated at 4°C overnight. Cells were washed 2X in staining buffer, then counted and following the last wash, 1 x 10^6^ cells were suspended in 100 µL of de-ionized water overnight. Time-of-flight mass spectrometry measured multiple different cellular parameters simultaneously in each cell. The percentage of AML stem cells (CLEC12A Hi, CD123 Hi, CD99 Hi, CD33 Hi, CD11b Lo) versus total cells in each sample and absolute fold-change of protein expression changes in ziftomenib-treated over control cells within the CLEC12A Hi, CD123 Hi, CD99 Hi, CD33 Hi, CD11b Lo population was analyzed by the Astrolabe Cytometry Platform (Astrolabe, Fort Lee, NJ).

**In vivo models of de novo AML:** All in vivo studies were approved by and conducted in accordance with the guidelines of the IACUC at the M.D. Anderson Cancer Center, an AAALAC-accredited facility. Male and female NOD.Cg-Prkdc^scid^ Il2rg^tm1Wjl^/SzJ (NSG) mice (stock number: 005557; 4-6 weeks of age) [Jackson Labs, Bar Harbor, ME; RRID: IMSR_JAX:005557] were exposed to 2.5 Gy of gamma radiation from a Cesium source. The following day, mice (n=10 per cohort) were injected in the lateral tail vein with 3.0 x 10^6^ luciferase-expressing AML PDX (Dana Farber PDX number: DF68555) cells and monitored for 5-7 days. Mice were imaged utilizing a Xenogen IVIS Lumina in vivo imaging system to document engraftment before treatment was initiated. Mice were randomized into groups based on equivalent mean bioluminescent intensity to control for variation in cell engraftment and variation between different treatment groups. Treatments were initiated on day 7. Mice were treated with ziftomenib (75 mg/kg, daily x 5 days, by oral gavage) and/or venetoclax (30 mg/kg, daily x 5 days, by oral gavage) or OTX015 (30 mg/kg, daily x 5 days, by oral gavage) for 6 weeks. Ziftomenib was prepared according to the reconstitution protocol provided by Kura Oncology. Venetoclax and OTX015 were prepared in a solution of 10% vol/vol of 95% ethanol, followed by 30% vol/vol of PEG-400, and then 60% vol/vol of Phosal-50. Mice were imaged weekly by bioluminescent imaging to document treatment efficacy and/or disease progression. Total bioluminescent flux was recorded as photons/second. Mice that became moribund or experienced hind limb paralysis were euthanized according to the approved IACUC protocol. Department of Veterinary Medicine staff members assisting in determining when euthanasia was required were blinded to the experimental conditions of the study. The survival of the mice is represented by a Kaplan-Meier plot. Significance was determined by a Mantel-Cox log rank test. P-values of less than 0.05 were assigned significance.

**Power analysis for in vivo studies**. With a sample size of 10 mice per group, we can achieve 79.5% power to detect a difference of overall survival at a significance level of 0.05 with one-sided log-rank test, assuming 30% of mouse-survival at the end of study in the experimental group.

**Statistical analysis**. Significant differences between values obtained in AML cells treated with different experimental conditions compared to untreated control cells were determined using the Student’s t-test in GraphPad V9. For the *in vivo* mouse models, a two-tailed, unpaired t-test was utilized for comparing total bioluminescent flux. For survival analysis, a Kaplan-Meier plot and a Mantel–Cox log rank test were utilized for comparisons of different cohorts. P-values < 0.05 were assigned significance.

**REFERENCES for Supplemental Methods**

1. Khan M, et al. Clinical outcomes and co-occurring mutations in patients with runx1-mutated acute myeloid leukemia. Int J Mol Sci. 2017; 18: 1618.
2. Labun K, Montague TG, Gagnon JA, Thyme SB, Valen E. CHOPCHOP v2: a web tool for the next generation of CRISPR genome engineering. Nucleic Acids Res. 2016; 44: W272-6.
3. Ianevski A, He L, Aittokallio T, Tang J. SynergyFinder: a web application for analyzing drug combination dose-response matrix data. Bioinformatics. 2017; 33: 2413-2415.
4. Ianevski, A., Giri, A. K., and Aittokallio, T. SynergyFinder 2.0: visual analytics of multi-drug combination synergies, Nucleic Acids Res. 2020; 48: W488-W493.
5. Fiskus W, Verstovsek S, Manshouri T, Rao R, Balusu R, Venkannagari S, et al. Heat shock protein 90 inhibitor is synergistic with JAK2 inhibitor and overcomes resistance to JAK2-TKI in human myeloproliferative neoplasm cells. Clin Cancer Res. 2011;17: 7347-58.
